# Initial Pose Estimation Method for Robust LiDAR-Inertial Calibration and Mapping

**DOI:** 10.3390/s24248199

**Published:** 2024-12-22

**Authors:** Eun-Seok Park, Saba Arshad, Tae-Hyoung Park

**Affiliations:** 1 Department of Intelligent Systems & Robotics, Chungbuk National University, Cheongju 28644, Republic of Korea; 2 School of Electrical and Computer Engineering, Chungbuk National University, Cheongju 28644, Republic of Korea; sabarshad1000@gmail.com

**Keywords:** pose estimation, LiDAR-IMU calibration, handheld device, 3D LiDAR

## Abstract

Handheld LiDAR scanners, which typically consist of a LiDAR sensor, Inertial Measurement Unit, and processor, enable data capture while moving, offering flexibility for various applications, including indoor and outdoor 3D mapping in fields such as architecture and civil engineering. Unlike fixed LiDAR systems, handheld devices allow data collection from different angles, but this mobility introduces challenges in data quality, particularly when initial calibration between sensors is not precise. Accurate LiDAR-IMU calibration, essential for mapping accuracy in Simultaneous Localization and Mapping applications, involves precise alignment of the sensors’ extrinsic parameters. This research presents a robust initial pose calibration method for LiDAR-IMU systems in handheld devices, specifically designed for indoor environments. The research contributions are twofold. Firstly, we present a robust plane detection method for LiDAR data. This plane detection method removes the noise caused by mobility of scanning device and provides accurate planes for precise LiDAR initial pose estimation. Secondly, we present a robust planes-aided LiDAR calibration method that estimates the initial pose. By employing this LiDAR calibration method, an efficient LiDAR-IMU calibration is achieved for accurate mapping. Experimental results demonstrate that the proposed method achieves lower calibration errors and improved computational efficiency compared to existing methods.

## 1. Introduction

As the demand for Light Detection and Ranging (LiDAR) continues to rise, handheld LiDAR devices that allow users to capture data while moving are becoming increasingly popular alongside fixed LiDAR systems mounted on autonomous vehicles. These handheld LiDAR devices are lightweight and enable users to freely acquire data on the go, thus making them suitable for various applications such as indoor and outdoor 3D scanning in fields like architecture and civil engineering [1]. A typical handheld LiDAR scanner consists of a LiDAR sensor, an Inertial Measurement Unit (IMU), camera, and a processor. Compared to fixed LiDAR systems, handheld LiDAR offers the advantage of greater flexibility, as it can collect data from various angles and positions. However, this high degree of freedom leads to decline in data quality if the initial calibration between sensors is not precise [2].

Handheld scanners typically employs Simultaneous Localization and Mapping (SLAM) technology to generate maps and estimate the robot position in GPS-denied environments [3,4,5]. The generated map allows the system to accurately determine its location within the environment, plan movement paths, and understand its surroundings during navigation. Therefore, environment mapping plays a critical role in the performance of SLAM-based autonomous navigation, making the creation of precise maps a crucial task. To generate such precise maps, accurate calibration of the extrinsic parameters between the LiDAR and the IMU is essential.

**Figure 1:**
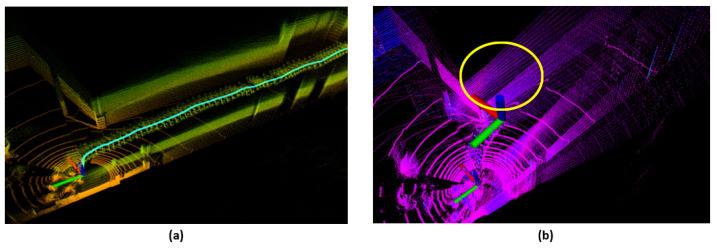
LiDAR based mapping using (**a**) LiDAR-IMU calibration method: Error-free mapping, and (**b**) Without LiDAR-IMU calibration method: Mapping error due to drift, highlighted in yellow circle. The colors in each map represents the intensity of LiDAR point cloud.

The LiDAR-IMU calibration refers to the process of precisely aligning the relative position and orientation between the LiDAR and IMU sensors [6,7,8,9,10,11,12,13]. While LiDAR provides precise recognition of the surrounding environment’s structure, its data can become distorted during high-speed movements or rapid motion. IMU, on the other hand, can accurately track short-term movements, but its errors tend to accumulate over time. By combining the strengths of these two sensors LiDAR-Inertial SLAM performance can be optimized. However, if the extrinsic parameters between the two sensors are not accurately calibrated, the data from both sensors may not align properly, causing distortions in the mapping process, which ultimately degrades the overall performance of system, as illustrated in Figure 1.

To improve the performance of LiDAR-IMU calibration, it is crucial to calibrate the initial pose of the LiDAR and IMU. Initial pose calibration refers to the process of calculating and adjusting the actual tilt angles of sensors based on the global coordinate system, rather than their local sensor coordinate frames. If the initial pose of LiDAR and IMU is not calibrated, the two sensors may have different tilts, which can lead to inaccurate data fusion. This may increase errors in position estimation or mapping processes. In particular, since IMU rotation data changes rapidly over short periods, failing to calibrate the initial pose can result in significant distortion in rotational measurements. This can negatively affect the overall system performance, making it essential to accurately calibrate the initial pose of both the LiDAR and IMU.

The existing research on LiDAR-IMU calibration used a pre-defined map [7], LiDAR scans [8], or ground information [10] for initial pose estimation. Though these approaches perform well in fixed LiDAR systems, the pose estimation accuracy is significantly low for moving platforms. Thus, this makes them unsuitable for handheld scanning systems.

In this research, we present a robust initial pose estimation method for LiDAR-IMU calibration on a handheld scanning system. The proposed method is designed for accurate mapping in an indoor environment. This is achieved by detecting robust planes in indoor environments and calibrating the initial poses of LiDAR and IMU based on the detected planes. To detect planes, we first divide the input data into voxels, and extract plane features from each voxel. Subsequently, we compute a plane score for each voxel using variance of the normal vectors and the point-to-plane distance. This allows for the robust detection of planes, even in the presence of edges and noise. The initial poses of the LiDAR and IMU are then calibrated based on the relationship between the detected planes and the actual planes. Through evaluation, it is shown that the proposed method achieves lower calibration errors compared to existing methods. Furthermore, the plane detection results used for calibration demonstrate improved performance over conventional plane detection algorithms.

The contributions of this paper are as follows.

- We propose a plane detection method based on plane scores; we can robustly detect planes effectively, even in the presence of noise and edges.
- We present an initial pose estimation method for LiDAR-IMU calibration. Using the relationship between the detected planes and the actual planes, stable calibration is achieved even under various movements.
- The proposed method demonstrates high calibration performance compared to existing methods on the benchmark dataset. The plane detection approach also proves to have higher accuracy and faster computational speed when compared to conventional algorithms.

## 2. Related Works

This section presents a critical review of the existing LiDAR-IMU calibration methods proposed for LiDAR-Inertial fusion SLAM systems. Based on the initial pose estimation strategy, existing methods are grouped into two categories; calibration methods with and without initial pose estimation. Each of these categories are explained below. Moreover, Table 1 presents a taxonomy of the existing research highlighting important characteristics for each method including the modalities, environments, and approach used for initial pose estimation and their limitations.

### 2.1. Methods Without Initial Pose Estimation

Das et al. [11] presented a method that dynamically calibrates the LiDAR sensor based on IMU data. The proposed method first estimates the position and orientation of the IMU relative to the vehicle frame. Then, using the calibrated pose of IMU, it calibrates the pose of the LiDAR relative to the vehicle frame. Though it has the advantage of performing real-time alignment between the two sensors using IMU data, it is sensitive to IMU drift and noise.

Lv et al. [12] estimated initial extrinsic parameters using LiDAR and IMU, extracted planes from LiDAR data, and performed optimization to achieve more accurate extrinsic calibration. This method has the advantage of not requiring a target and being robust against LiDAR motion distortion, yet it requires high computational resources, limiting its applicability in real-world environments. In [13], the authors proposed a method that estimates the initial extrinsic parameters between the two sensors and refines these parameters through iterative alignment. While this method has the advantage of being applicable in various environments, it does not perform initial pose estimation, like [12], and performed online but has the drawback of increased computational cost due to the iterative alignment process, making real-time processing challenging.

### 2.2. Methods with Initial Pose Estimation

Several studies have proposed LiDAR-IMU calibration methods to improve calibration accuracy by utilizing LiDAR data to accurately calibrate the LiDAR-IMU pose.

Le et al. [6] proposed a targetless-based approach for LiDAR-IMU calibration without the aid of other sensors. It corrects temporal motion distortion in the IMU by upsampling LiDAR data. Although the proposed method uses IMU data to correct distortions in LiDAR data, precise calibration is not achievable due to the use of low-accuracy upsampled LiDAR points. Furthermore, by relying solely on IMU data without the structural information of LiDAR, it focuses on motion distortion.

Li et al. [7] improved accuracy by correcting the IMU’s rotation and displacement based on LiDAR data and a pre-defined map containing structural environmental information. The proposed method allows for relatively easy estimation and correction of orientation by utilizing a pre-defined map. However, its computational complexity is very high due to time synchronization process performed through continuous path estimation. Also, this approach cannot be used if the predefined map is unavailable or inaccurate.

In [8], the authors presented a method that estimates the initial pose of LiDAR and IMU sensors using LiDAR data, robustly calibrated against disturbances in dynamic environments. This method firstly extracts planes from the first LiDAR scan. Secondly, the point-to-plane distance is calculated with planes extracted from subsequent LiDAR scans to correct the initial orientation of the LiDAR. This method is designed for real-time operation, addressing the computational complexity problem in [7]. However, its performance may vary depending on different environments. Also, this method relies solely on the relative relationship between data estimated within the LiDAR frame and does not consider direct alignment with the global frame. As a result, the accuracy of IMU calibration may degrade in case of significant movement or rotation.

Li et al. [9] proposed a method that performs spatial and temporal calibration of the IMU by not only utilizing LiDAR data but also visual information from camera. This method calibrates the IMU in two stages, thus enabling more precise estimation. However, the simultaneous use of LiDAR and camera requires a complex processing workflow, and the interdependencies between stages result in inconsistent performance.

Kim et al. [10] performed IMU calibration by incorporating LiDAR data and planar motion constraints, allowing calibration without a target and enabling simpler calibration through these constraints. This method extracts ground data from LiDAR mounted on a ground robot and calibrates the pose of the LiDAR and IMU by calculating the relationship between detected and the actual ground. Unlike [8], this method aligns directly with the global frame to calibrate the pose of the LiDAR and IMU, demonstrating improved calibration performance. However, for handheld LiDAR, which cannot always observe the ground due to device movement, relying solely on ground observations for calibration will affect accuracy on large scale.

In other words, the existing method lacks versatility or is unsuitable for use with portable LiDAR due to constraints such as reliance on predefined maps or ground information. While there are methods without such constraints, they have the drawback of not directly calibrating the initial pose between the global and local coordinate systems, making it challenging to ensure consistent performance. This paper aims to overcome the limitations of previous studies and proposes an initial pose estimation method for accurate calibration of LiDAR-IMU embedded in handheld scanning systems. For this purpose, we firstly extract plane information from the real environment in a global frame, then we estimate the orientation of LiDAR using the relationship between global and LiDAR coordinates, and lastly, we use LiDAR orientation to estimate the IMU orientation which enables LiDAR-IMU calibration without complex procedures. The propose method is designed to ensure its application in highly dynamic environments.

## 3. Method Overview

### 3.1. System Architecture

**Figure 2:**
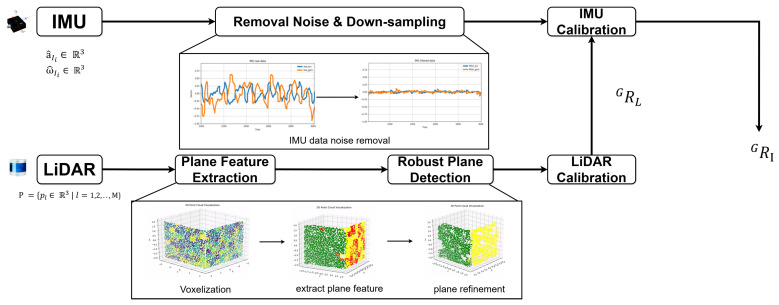
Overall framework of the proposed initial pose estimation method for robust LiDAR-IMU calibration. Different colors in voxelization shows the intensity of LiDAR points in each voxel. The extracted planes are represented with yellow and green color while red color points indicate noise.

This paper focuses on calibrating the initial pose of a handheld LiDAR and IMU. The overall pipeline of the proposed framework is shown in Figure 2. Firstly, plane features are extracted using the input LiDAR data. Then, these plane features are given as input to the Robust Plane Detection module, which detects the planes that are resilient to edges and noise. Using those planes, the initial pose of LiDAR is calibrated. On the other hand, noise is removed from the input IMU measurements. Finally, the initial pose of IMU is calibrated using IMU measurements and LiDAR calibration.

### 3.2. Notations

Several important notations used in this paper are presented in Table 2. The LiDAR frame and IMU frame refer to the coordinate systems relative to the LiDAR and IMU, respectively, while absolute frame represents the global coordinate system.

## 4. Proposed Method

### 4.1. Plane Feature Extration

For plane feature extraction, the input LiDAR data *P* is divided into W×H×C voxels. Then, the normal vector is repeatedly calculated *N* times for each voxel, $v_{w,h,c}$ located at position (w,h,c) and the average normal vector ${\bar{n}}_{w,h,c}\in R^{3}$ is computed using Equation (1):

(1) $${\bar{n}}_{w,h,c}=\left(\frac{1}{N}\sum_{j=1}^{N}n_{x_{w,h,c}}^{\left(j\right)},\frac{1}{N}\sum_{j=1}^{N}n_{y_{w,h,c}}^{\left(j\right)},\frac{1}{N}\sum_{j=1}^{N}n_{z_{w,h,c}}^{\left(j\right)}\right)$$

where $n_{x_{w,h,c}}^{j},n_{y_{w,h,c}}^{j},n_{z_{w,h,c}}^{j}$ refer to the x,y,z components of the *j*-th normal vector $n_{w,h,c}^{j}$ within voxel $v_{w,h,c}$. Consequently, each voxel $v_{w,h,c}$ contains total *N* normal vectors and one average vector ${\bar{n}}_{w,h,c}$, expressed as $v_{w,h,c}=\left\{{\bar{n}}_{w,h,c},n_{w,h,c}^{1},n_{w,h,c}^{2},\cdots ,n_{w,h,c}^{N}\right\}$.

### 4.2. Robust Plane Detection

**Figure 3:**
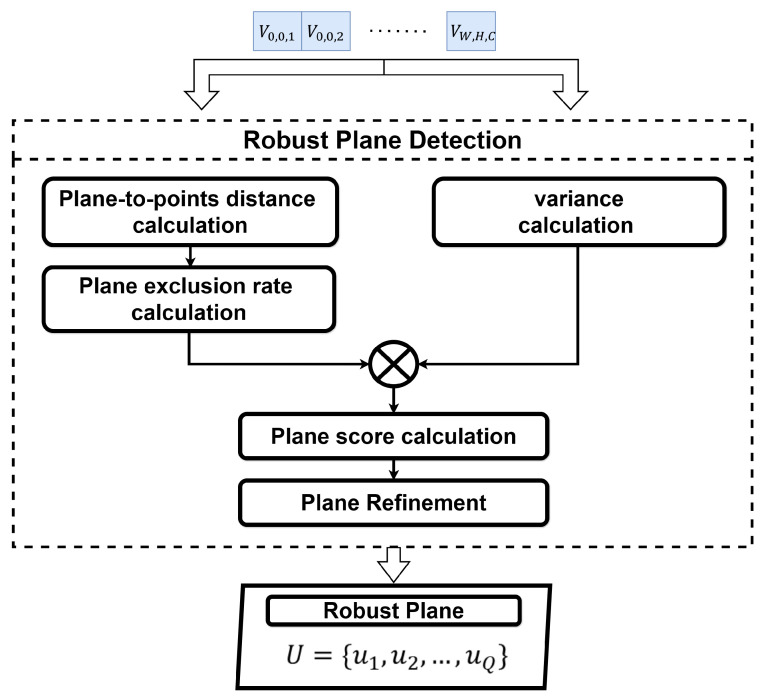
Robust plane detection method.

This module takes normal vectors as input and detects robust planes using the W×H×C set of voxels $V_{W,H,C}$ as $V_{W,H,C}=\{v_{w,h,c}|w=1,2,\cdots ,W\;;\;h=1,2,\cdots ,H\;;$c=1,2,⋯,C}, which includes the *N* normal vectors and the average vector ${\bar{n}}_{w,h,c}$ calculated for each voxel. The configuration for plane detection is shown in Figure 3. For robust plane detection, this module computes a plane score utilizing the variance of normal vectors $n_{w,h,c}^{1,\ldots ,N}$ and point-to-plane distance.

The variance of normal vectors represents the directionality of the normal vectors detected in the current voxel. In general, planes in indoor environments have flat structures. However, near edges or boundaries, where two or more different planes intersect, the normal vectors in this region can point in multiple directions. In other words, while calculating the normal vectors for each voxel, the normal vectors in the planar region tend to have almost the same direction, whereas the regions containing edges may have normal vectors pointing in various directions. To ensure the consistency of the normal vector directions within a voxel $v_{w,h,c}$, the variance $\sigma^{2}$ of the normal vectors $n_{w,h,c}^{\left(1,\ldots ,N\right)}$ given as $\sigma_{n_{w,h,c}}^{2}$ is calculated using Equation (2):

(2) $$\sigma_{n_{w,h,c}}^{2}=\frac{1}{N}\sum_{j=1}^{N}{∥r_{w,h,c}^{\left(j\right)}∥}^{2}=\frac{1}{N}\sum_{j=1}^{N}{∥n_{w,h,c}^{\left(j\right)}-{\bar{n}}_{w,h,c}∥}^{2}$$

where ${∥r_{w,h,c}^{\left(j\right)}∥}^{2}\in R$ denotes the deviation between *j*-th normal vector $n_{w,h,c}^{\left(j\right)}$ and the average vector ${\bar{n}}_{w,h,c}$ of voxel. A planar region with consistent vector directions will have low variance, while an edge region with vectors pointing in multiple directions will exhibit high variance.

Next, the point-to-plane distance is calculated, to mitigate the impact of noise. The point-to-plane distance determines how well the given points align with a specific plane. The distance $d_{i}$ between the *i*th point in voxel $v_{w,h,c}$ and the plane ${\bar{n}}_{w,h,c}$ is computed using Equation (3):

(3) $$\begin{array}{c} d_{i}=\frac{|{\bar{n}}_{x_{w,h,c}}x_{i}+{\bar{n}}_{y_{w,h,c}}y_{i}+{\bar{n}}_{z_{w,h,c}}z_{i}+D|}{\sqrt{{\bar{n}}_{x_{w,h,c}}^{2}+{\bar{n}}_{y_{w,h,c}}^{2}+{\bar{n}}_{z_{w,h,c}}^{2}}} \end{array}$$

where *D* represents the distance of the plane from origin in LiDAR coordinate system. The larger the point-to-plane distance is the more will be the noise. Thus, among all the points $P_{w,h,c}^{\prime }$ in a voxel, we select the points $p_{v_{w,h,c}}^{i,\ldots ,K}$ with $d_{i}$ less than certain threshold $d_{thresh}$, while discarding those with higher distance, as given in Equation (4):

(4) $${\mathrm{Num}}_{pt}=\left\{p_{v_{w,h,c}}^{i,\ldots ,K}\in P_{w,h,c}^{\prime }|d_{i}<d_{thresh}\right\}$$

where ${\mathrm{Num}}_{pt}$ represents the set of robust points in a voxel $v_{w,h,c}$. These robust points are used to compute the plane score $S_{w,h,c}$ for each voxel, using Equation (5):

(5) $$\begin{array}{cc} S_{w,h,c} & =\left(1-\sigma_{n_{w,h,c}}^{2}\right)\times \frac{{\mathrm{Num}}_{pt}}{{\mathrm{Num}}_{P_{w,h,c}^{\prime }}} \end{array}$$

where the value $S_{w,h,c}$ ranges between 0 and 1 and ${\mathrm{Num}}_{P_{w,h,c}^{\prime }}$ represents the number of total points contained within $v_{w,h,c}$.

Once the plane scores are computed, plane refinement is performed, which further reduces the noise by eliminating the noisy voxels. For each voxel $v_{w,h,c}$, plane score $S_{w,h,c}$ is compared with a predefined threshold $s_{thresh}$. If $S_{w,h,c}<s_{thresh}$ the plane score for $v_{w,h,c}$ is redefined using the neighboring voxels, $V_{negibor}$. This score redefining is achieved by computing the distance $d_{k}$ between each point $p_{w,h,c}$ in $v_{w,h,c}$ with average normal vector ${\bar{n}}_{w,h,c}$ of neighboring voxels using Equation (3).

**Figure 4:**
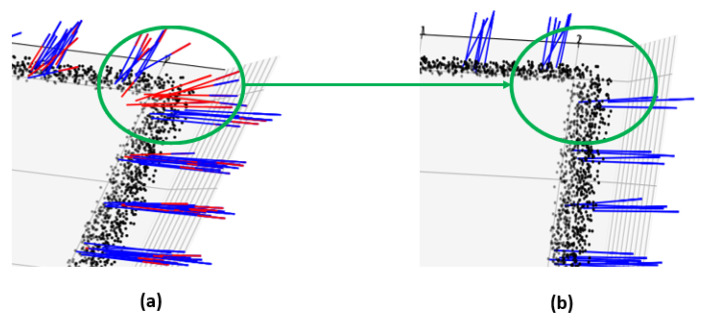
Robust plane extraction through refinement. (**a**) Voxels containing edges and noise have low plane scores due to large distances and high variance represented as red color normal vector while those with high plane scores are represented with blue. (**b**) The refinement process enables the effective separation and removal of areas containing edges and noise.

If the distance $d_{k}<d_{thresh}$, $p_{w,h,c}$ is associated with the relevant neighboring voxel, while if $d_{k}>d_{thresh}$, $p_{w,h,c}$ is recognized as a noise and is simply discarded. This allows for noise removal and edge correction for voxels with low plane scores $S_{w,h,c}$. Figure 4 depicts the plane refinement process. The final detected robust planes are denoted as $u_{q}\in U$, with a total of *Q* planes and q=1,2,…Q. Here, $u_{q}$ represent each plane with plane score $s_{q}$ and normal vector $n_{q}$. The complete process of robust plane detection is explained in Algorithm 1.
**Algorithm 1** Robust Plane Detection Algorithm**Input: **$V_{W,H,C}$: Input Voxel, $s_{th}$: score threshold, $d_{th}$: refine threshold**Output: ***U*: Detected plane**for **$w,h,c\in V_{W,H,C}$ **do**    ▹ Iterate over all voxels    $\sigma_{n_{w,h,c}}^{2}\leftarrow \frac{1}{N}\sum_{j=0}^{N-1}||n\left[j\right]-\bar{n}\left|\right|$    $rate\leftarrow \frac{1}{|P_{w,h,c}|}\sum_{p\in P_{w,h,c}}⊮\left(\mathrm{distance}\left(p,\bar{n}\right)<d_{thresh}\right)$    $S_{w,h,c}\leftarrow \left(1-\sigma_{n_{w,h,c}}^{2}\right)\times rate$    **if** $S_{w,h,c}<s_{th}$ **then**        **for** *i* in range [w−1,w+1] **do**           **for** *j* in range [h−1,h+1] **do**               **for** *k* in range [c−1,c+1] **do**                   $V_{neighbor}\leftarrow V_{i,j,k}$               **end for**           **end for**        **end for**        **for** $pt\in P_{w,h,c}$ **do**           **for** $v_{neighbor}\in V_{neighbor}$ **do**               Compute $dist\leftarrow \mathrm{distance}(pt,v_{neighbor}.\bar{n})$               **if** $dist<d_{th}$ **then**                   $P_{neighbor}\leftarrow P_{neighbor}\cup \left\{pt\right\}$               **end if**           **end for**        **end for**    **else**        $U\leftarrow U\cup P_{w,h,c}$    **end if****end for**

### 4.3. LiDAR Calibration

**Figure 5:**
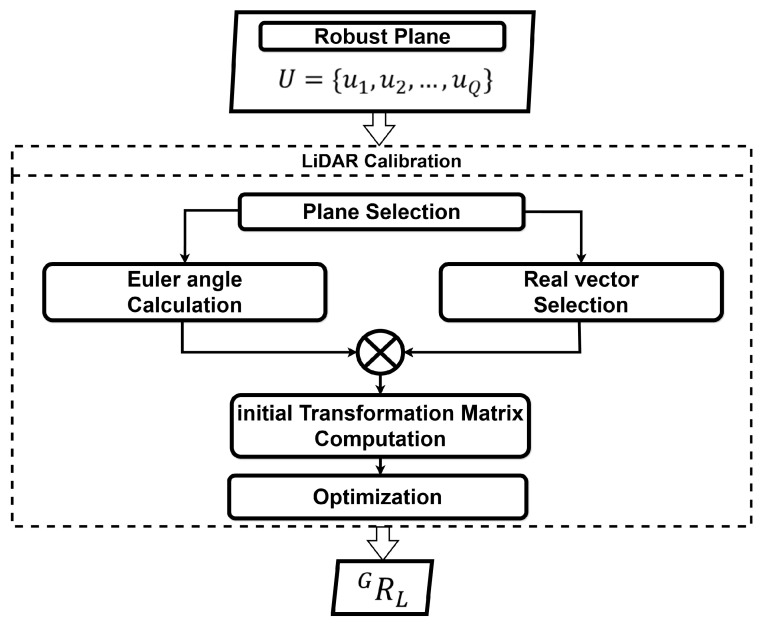
LiDAR calibration method.

The calibration of the LiDAR’s initial pose refers to the process of calculating a transformation matrix based on the relationship between the LiDAR frame and the global frame. Planes are generally defined with respect to the global coordinate system. However, the LiDAR coordinate system is based on the LiDAR itself. Therefore, if the LiDAR sensor is tilted, even a perfectly flat plane will appear as an inclined plane in the LiDAR coordinate system. As a result, the LiDAR coordinate system is not aligned with the global coordinate system. This paper estimates the initial pose of the LiDAR to address the misalignment between these two coordinate systems. The detected robust planes $u_{q}$, including a plane score $s_{q}$ and normal vectors $n_{q}$, are given as input to the LiDAR calibration method, as depicted in Figure 5.

The initial pose LiDAR calibration is based on the following two assumptions:

- The indoor spaces have a cuboidal structure and are composed of at least two or more planes, including walls, floors, and ceilings;
- Each plane is flat and the angles between planes are perpendicular. Unlike outdoor environments, the planes that make up indoor spaces, such as walls, floors, and ceilings, are flat and each plane is oriented perpendicular to its neighboring planes.

Based on the aforementioned assumptions, we define the normal vectors of the planes that constitute indoor spaces in real environments, as given in Equation (6):

(6) $$n_{G}=\left\{\left[\begin{array}{c} 0\\ 0\\ 1 \end{array}\right],\left[\begin{array}{c} 0\\ 1\\ 0 \end{array}\right],\left[\begin{array}{c} 1\\ 0\\ 0 \end{array}\right],\left[\begin{array}{c} 0\\ 0\\ -1 \end{array}\right],\left[\begin{array}{c} 0\\ -1\\ 0 \end{array}\right],\left[\begin{array}{c} -1\\ 0\\ 0 \end{array}\right]\right\}$$

where $n_{G}$ is a set of plane vectors $n_{g}$, each representing the real environment including walls and ceilings in an indoor setting. Positive and negative values represent directions, where a vector with a negative value indicates the opposite direction of a vector with a positive value. We define $n_{G}$ as the global frame and compute the transformation with respect to the detected planes *U*.

Handheld LiDAR has greater degrees of freedom compared to ground robots, which are limited to planar motion. Specifically, the yaw motion of handheld LiDAR is similar to that of fixed LiDAR mounted on ground robots; it exhibits a larger range of motion in the roll and pitch axes. This paper emphasizes the correction of the roll and pitch axes among the three rotational axes. Additionally, this paper highlights the issue of non-linear tilting of sensors caused by the high degree of freedom in handheld LiDAR systems. This means that while the sensor’s position may be accurate, its rotational pose may not be properly aligned. Therefore, this study focuses solely on the calibration of the sensor pose and does not consider positional information. The sensor’s position is fixed at global frame origin O(0,0,0), and the rotation matrix between the global frame $n_{G}$ and LiDAR frame is calculated through optimization.

To calibrate LiDAR pose orientation, the two planes with the highest plane scores are used. After projecting these two planes in the same direction, an average vector $n_{avg}$ is computed. In this paper, the rotation matrix is calculated using vectors, so it is not necessary for the two vectors to be orthogonal to each other. Therefore, when the angle between the two vectors is close to perpendicular, two vectors are projected in the same direction to make them parallel, and then their average vector is calculated. In this case, the projection rotates 90 degrees based on the vector with the higher plane score. If the two vectors are parallel to each other, the average vector of the two is calculated. The average vector $n_{avg}$ is a plane vector used to estimate the current pose of the LiDAR frame. The normal vector between two frames is computed and is used as the initial calibration matrix $R_{init}$:

(7) $$\begin{array}{c} \begin{array}{c} n=\arg\min_{n_{g}\in n_{G}}\left[\cos^{-1}\left(\frac{n_{avg}\cdot n_{g}}{∥n_{avg}∥∥n_{g}∥}\right)\right] \end{array} \end{array}$$

(8) $$\begin{array}{c} R_{\mathrm{init}}=\frac{n\cdot n_{avg}^{T}}{n_{avg}\cdot n_{avg}^{T}} \end{array}$$

where *n* refers to the vector $n_{g}$ within $n_{G}$ that has the smallest angle with $n_{avg}$. Based on this initial rotation matrix $R_{\mathrm{init}}$, optimization is performed to correct the pose in LiDAR frame to the global frame. The cost function is computed as follows:

(9) $$\begin{array}{c} {\mathrm{loss}}_{q}={∥Rn_{q}-n_{g}∥}^{2} \end{array}$$

where ${\mathrm{loss}}_{q}$ represents the cost function, where R∈SO(3) denotes the rotation matrix that changes through optimization, with $R_{\mathrm{init}}$ as the initial value. The ${\mathrm{loss}}_{q}$ is calculated based on the deviation between the robust plane vector $n_{q}$, calibrated by *R*, and $n_{g}$.

The Transformation matrix *R* is optimized by using ${\mathrm{loss}}_{q}$ and score $s_{q}$ for each plane, as depicted in Equation (10):

(10) RLG=argminR∑q=1Q(1−sq)·lossq

The LiDAR calibration is explained in Algorithm 2.
**Algorithm 2** LiDAR Calibration Algorithm**Input:** $U_{Q}$: Detected planes**Output:** RLG: Optimized rotation matrix **Step 1: Sort and Select Planes** Sort $U_{Q}$ in descending order based on scores Select the top two planes: $P_{1}\leftarrow U_{Q}\left[0\right]$, $P_{2}\leftarrow U_{Q}\left[1\right]$ **Step 2: Compute Initial Transformation $R_{init}$** Project $P_{2}$ onto $P_{1}$ and compute their mean $n_{avg}$ Compute rotation matrix: $R_{init}=n_{avg}^{-1}n_{g}$ **Step 3: Optimize the Transformation Matrix** Initialize $R\leftarrow R_{init}$ **while** not converged **do**     Compute loss:
${\mathrm{loss}}_{P}={∥n_{g}-R\cdot P∥}^{2}$     Update transformation matrix:
RLG=argminR∑P∈UQ(1−SP)∗lossP **end while**

### 4.4. IMU Noise Removal, Downsampling, and Calibration

For IMU pose correction and calibration, a method similar to the existing approach [10] is used. The IMU measures acceleration ${\hat{a}}_{i}$ and angular velocity ${\hat{\omega }}_{i}$ at each time point *i*. The IMU measurement model is given in Equations (11) and (12):

(11) $$\begin{array}{c} {\hat{a}}_{i}=a_{i}+R_{i}^{w}g^{w}+b_{a}+\epsilon_{a} \end{array}$$

(12) $${\hat{\omega }}_{i}=\omega_{i}+b_{g}+\epsilon_{g}$$

where $a_{i}$ and $\omega_{i}$ are the ground truth of IMU angular velocity and linear acceleration, $R_{i}^{w}$ is a matrix that transforms the gravity vector into the IMU frame, $b_{a}$ and $b_{g}$ denote the biases for linear acceleration and angular velocity, and $\epsilon_{a}$ and $\epsilon_{g}$ represent the Gaussian noise for acceleration and angular velocity, respectively.

**Figure 6:**
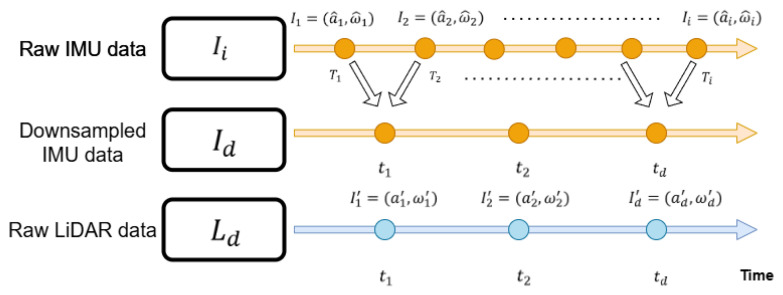
IMU downsampling.

Generally, the sampling rate of IMU data is higher than that of LiDAR data. Moreover, downsampling of the IMU data is performed as shown in Figure 6. Equations (13) and (14) are used to compute the downsampled acceleration $a_{d}^{\prime }$ and angular velocity $\omega_{d}^{\prime }$:

(13) $$a_{d}^{\prime }=\left(\frac{1}{C}\sum_{i=1}^{C}{\hat{a}}_{i}^{x},\frac{1}{C}\sum_{i=1}^{C}{\hat{a}}_{i}^{y},\frac{1}{C}\sum_{i=1}^{C}{\hat{a}}_{i}^{z}\right)$$

(14) $$\omega_{d}^{\prime }=\left(\frac{1}{C}\sum_{i=1}^{C}{\hat{\omega }}_{i}^{x},\frac{1}{C}\sum_{i=1}^{C}{\hat{\omega }}_{i}^{y},\frac{1}{C}\sum_{i=1}^{C}{\hat{\omega }}_{i}^{z}\right)$$

where *C* denotes the number of accumulated IMU data until one LiDAR scan is received. In other words, downsampling is performed by calculating the average of accumulated IMU data until a single LiDAR data point is acquired. Subsequently, the downsampled data are processed using Zero Phase Filter (ZPF) [14] to remove noise components, $\epsilon_{a}$ and $\epsilon_{g}$. ZPF typically utilizes a linear filter that considers both past and future data points to perform filtering.

Finally, the IMU acceleration and angular velocity data, with noise removed by the ZPF, are input into the Madgwick filter [15] to estimate the IMU pose at the current time. The Madgwick filter provides the current quaternion q = [$q_{w}$,$q_{x}$,$q_{y}$,$q_{z}$], which is then used to construct the rotation matrix RIG for calibration of the IMU pose.

## 5. Results

### 5.1. Experimental Setup

The experiments are conducted using a PC equipped with an Intel Core™ i7-12700KF CPU and an Nvidia GeForce RTX 3080 GPU. The proposed method is implemented in Ubuntu 20.04 environment, C++, OpenCV, Eigen, PCL (Point Cloud Library), and ROS1 libraries. In the first experiment, the accuracy of the proposed plane detection method for sensor calibration is evaluated. The evaluation metrics used in the experiment include Precision, Recall, and F1-score for the detected planes. In the second experiment, calibration results of the proposed method are compared to the existing methods, LI-init [8] and GRIL-Calib [10], using the Root Mean Squared Error (RMSE).

### 5.2. Datasets

The proposed method is evaluated on publicity available benchmark datasets, i.e, LiDAR-Net [16], Hilti dataset [17], and VECtor dataset [18]. LiDAR-Net [16] contains 3D LiDAR point clouds from various indoor environments, with detailed labeling for multiple objects and planes such as walls, ceilings, and floors. The Hilti [17] and VECtor [18] datasets are designed for SLAM, acquired using handheld LiDAR and ground robots in diverse indoor and outdoor environments. Each dataset includes both LiDAR and IMU data. Details of sensor models used in the acquisition of these datasets are listed in Table 3 and Table 4.

As can be seen in Table 4, while the IMUs included in both datasets contain accelerometer, gyroscope, and magnetometer sensors, this paper only utilized the accelerometer and gyroscope data.

### 5.3. Evaluation of Robust Plane Detection Method

This section present the performance results of the proposed robust plane detection method.

#### 5.3.1. Impact of Robust Plane Selection

Among all the detected planes, the robust plane detection module selects the robust planes while removing noise and discarding the corners. This plane selection involves point-to-plane distance $d_{i}$ computation between each *i*th point in voxel $v_{w,h,c}$ and plane ${\bar{n}}_{w,h,c}$. The robust plane includes the points with $d_{i}<d_{thresh}$, only removing the points with $d_{i}$ higher than $d_{thresh}$.

The higher the threshold, the more noisy and robust points the data contain, while lower threshold value results in removal of noise along with robust points. We have performed several experiments to find the optimal value for $d_{thresh}$. Table 5 lists the impact of threshold $d_{thresh}$ on the overall performance of the proposed method in terms of F1-score and computation time. Moreover, we have also analyzed the impact of sensor noise σ on overall performance in terms of F1 score and Time. Table 5 lists the results of $d_{thresh}$ for different values of Gaussian noise σ=0 (which represents no noise), σ=0.03, and σ=0.05, respectively.

According to the experimental results, the proposed method shows best performance for $d_{thresh}=0.2$. Setting $d_{thresh}$ too high, closer to 1, results in lower performance because it does not sufficiently remove the noise and corners. Conversely, setting $d_{thresh}$ too low, closer to 0, reduces the influence of noise but also removes the robust points, which leads to a decline in overall performance. It is also noted that sensor noise affects the performance of the system, yet the highest F1 score is still achieved using $d_{thresh}=0.2$. Therefore, this paper sets $d_{thresh}$ to 0.2.

#### 5.3.2. Plane Refinement Performance Analysis

Once the robust planes are selected, the plane refinement is performed to further remove the noise. The plane score $S_{w,h,c}$ for each voxel $v_{w,h,c}$ is computed and if the score is below certain threshold $s_{thresh}$, the plane score for $v_{w,h,c}$ is redefined. Planes with a score $S_{w,h,c}>s_{thresh}$ are considered final robust planes. Thus, the lower the threshold will be, the lower the number of planes that are redefined, and thus, the less noise is removed. On the other hand, higher threshold values, closer to 1, will not only remove noise, but will cause redefining of robust voxels, resulting in distortion of robust data. We have performed several experiments to find the optimal value for $s_{thresh}$ in order to achieve the best performance for plane refinement. Table 6 lists the impact of plane refinement on overall performance of the proposed method in terms of F1-score and computation time. Moreover, we have also analyzed the impact of sensor noise σ on overall performance in terms of F1 score and Time. Table 6 lists the results of $s_{thresh}$ for different values of Gaussian noise σ=0 (which represents no noise), σ=0.03, and σ=0.05, respectively.

It can be observed that setting the threshold $s_{thresh}=0.2$ achieves best performance. If $s_{thresh}$ is set too low, few or no planes are subject to refinement, which reduces overall accuracy as incorrect planes are not improved. Conversely, if $s_{thresh}$ is set too high, even correctly detected planes undergo refinement, potentially decreasing performance. It is also noted that sensor noise affects the performance of the system, yet the highest F1 score is still achieved using $s_{thresh}=0.2$. Therefore, this paper sets $s_{thresh}$ to 0.2.

#### 5.3.3. Comparison with Other Plane Detection Methods

The proposed robust plane detection method for initial pose calibration is evaluated and compared with other plane detection algorithms, such as RANSAC [19], Region Growing [20], and 3D-Hough Transform [21]. The experiments are conducted using room and corridor data included in the LiDAR-Net dataset. Although the dataset provides detailed labeling for planes, such as walls, ceilings, and floors, the proposed plane detection method does not classify the detected planes into these categories. Therefore, if the points of the detected plane match the labeled points of the walls, ceilings, or floors in the dataset, the True Positive (TP) count is increased. The results of the experiment are shown in Table 7, in terms of Precision, Recall, and F1-score.

The experimental results demonstrate that the proposed method achieves higher plane detection accuracy compared to existing methods. Specifically, the proposed method achieves noticeable improvement of up to 4.7% and 13.3% on the corridor and room sequence, in terms of F1-score. In terms of Recall, the proposed method achieves 12.7% and 18% improvement on the corridor and room sequences, respectively. Though RANSAC [19] and the 3D-Hough Transform [21] methods achieved higher precision, they had lower recall, resulting in lower F1-scores. This implies that while these methods had high prediction rates for data identified as planes, they struggle to accurately detect planes in the dataset.

**Figure 7:**
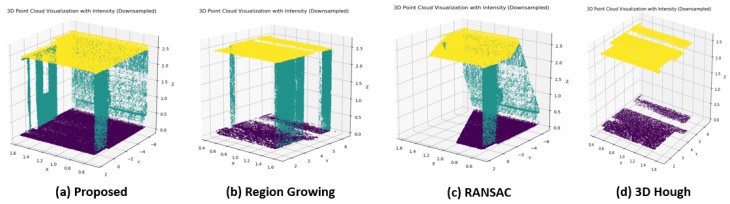
Qualitative Comparison of the proposed method with the benchmark plane detection algorithms.

The proposed method also detected planes with significantly faster computational speed compared to existing methods. The proposed method demonstrated approximately 101 times and 147 times faster computational speed than the Region Growing method [20] in the corridor and room environments, respectively, while being 1.41 times and 2.09 times faster, respectively, than RANSAC. The qualitative comparison of plane detection on corridor data are given Figure 7.

For visualization, points detected as planes by the algorithms are compared with the dataset labels, and if they corresponded to a wall, ceiling, or floor, the points are colored green, yellow, and purple, respectively. It can be observed that the proposed method can accurately detect planes compared to existing methods.

### 5.4. Evaluation of Initial Pose Estimation Methods

In this section, calibration accuracy is evaluated using the Hilti dataset [17] and VECtor dataset [18]. The evaluation method involves calculating odometry using LiDAR and IMU calibrated to the global frame based on the detected planes, followed by computing roll and pitch from the odometry. The roll and pitch errors are then calculated against the ground truth using the Root Mean Squared Error (RMSE) and compared with the existing methods LI-init [8] and GRIL-Calib [10]. The units are in degrees, with the ground truth set to 0°.

**Figure 8:**
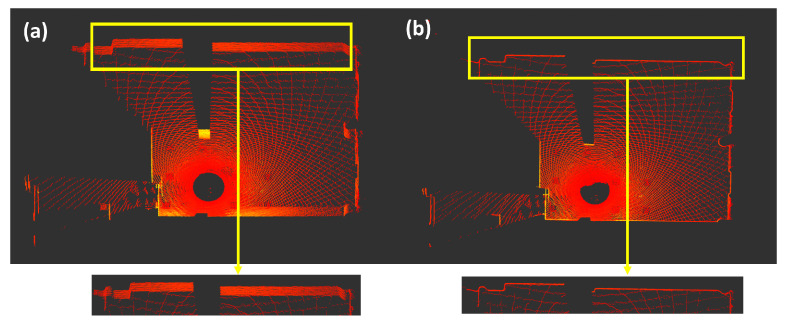
Top view of LiDAR data. (**a**) LiDAR raw data before calibration. (**b**) LiDAR data after calibration using the proposed method.

In LI-init [8], a handheld LiDAR is used, while GRIL-Calib [10] performs calibration using LiDAR mounted on a ground robot. Both of these methods are implemented using open-source software provided by the authors. Therefore, in this experiment, a comparative evaluation with existing methods is conducted using the data acquired from a ground robot in the Hilti dataset [17] and from a handheld LiDAR in the VECtor dataset [18]. Data were collected in the Basement and Corridor environments. The results of LiDAR calibration are shown in Figure 8, and the RMSE for roll and pitch are presented in Table 8.

Figure 8 shows the top view of the LiDAR data after calibrating the sensor’s pose using the rotation matrix computed by the proposed method. In the initial input data, the tilt of the sensor causes the wall to appear as multiple overlapping layers. However, after the proposed calibration process, the LiDAR data are accurately aligned, displaying the wall as a single, clean layer without any overlaps. This demonstrates that the sensor’s tilt and distortion have been effectively removed.

The proposed method did not show significant differences compared to existing methods for the Hilti dataset [17], which utilizes a ground robot. In fact, GRIL-Calib [10] demonstrated the best performance, likely due to its calibration approach, specifically designed for ground robots. However, in the case of the VECtor dataset [18], which uses a handheld LiDAR, the proposed method shows significantly lower calibration errors compared to the existing methods. This can be explained by the limitations of GRIL-Calib [10], which relies solely on ground data, resulting in reduced calibration accuracy when ground data are not detected by the LiDAR. On the other hand, LI-init [8] uses planar information for calibration but only considers the relative relationships within the LiDAR frame without considering direct alignment with the global frame, leading to a notable decrease in sensor calibration accuracy.

**Figure 9:**
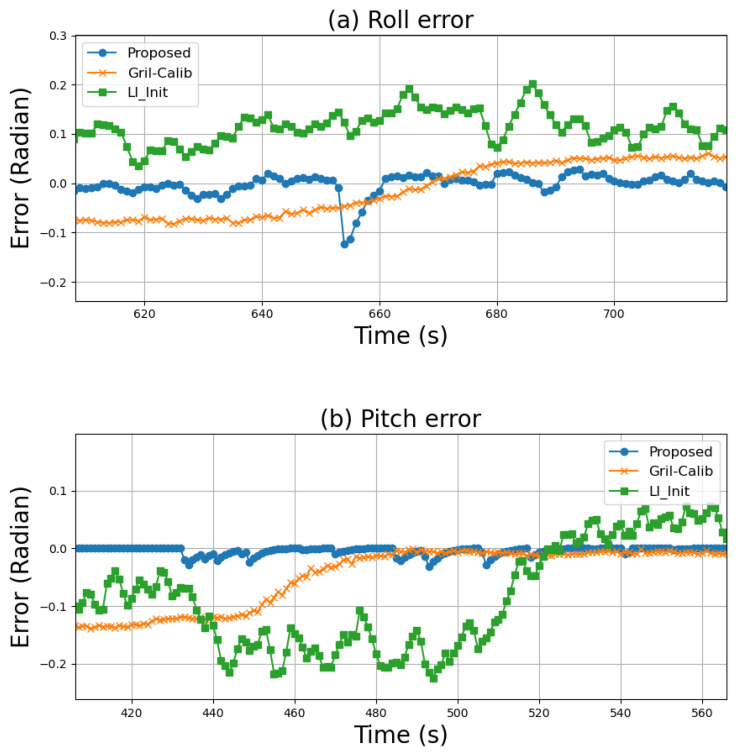
Performance comparison in terms of (**a**) roll and (**b**) pitch errors in the VECtor dataset.

The proposed method, as defined by using the normal vector of the real environment plane in the global frame, is defined in Equation (7), and calculating the relationship with the detected plane demonstrates better calibration performance compared to existing methods. Figure 9 shows the roll and pitch errors of the proposed and existing methods in the corridor environment. It is evident from the results that the proposed method outperforms the existing methods with lower calibration errors.

### 5.5. Evaluation of Mapping Result

**Figure 10:**
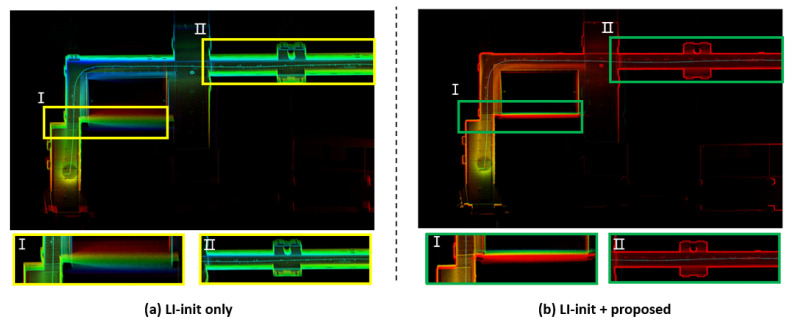
Performance comparison in terms of the (**a**) mapping result using LI-init and (**b**) mapping result using LI-init+Proposed.

This study focuses on the initial pose calibration of a moving handheld LiDAR and IMU system, rather than a fixed LiDAR. To evaluate the impact of the proposed initial pose estimation method on mapping, a comparison was made with existing approaches. Therefore, mapping was performed by replacing the initial pose estimation method used in LI-init, which performs both initial pose estimation and extrinsic parameter calibration for mapping, with the proposed initial pose estimation method. The results are shown in Figure 10.

Figure 10 shows the mapping results using only LI-init and the mapping results obtained by integrating LI-init with the proposed method. Since LI-init estimates the initial pose based on relative alignment between LiDAR scan data rather than with respect to the global frame, errors in initial pose estimation occur, leading to uncorrected sensor tilt and resulting in distortion in the map. However, in the map generated by integrating the proposed initial pose estimation method, it can be observed that the map is created without any distortion. Additionally, the color of the map represents the z-axis error of each point. In other words, the existing method shows significant z-axis errors due to incorrect initial pose estimation, while the map generated using the proposed method demonstrates relatively smaller z-axis errors.

## 6. Conclusions

This research addresses the problem of accurate initial pose estimation in handheld LiDAR-IMU systems, which are essential for creating precise maps in indoor environments. While handheld LiDAR scanners offer flexibility and portability, ensuring accurate data alignment between LiDAR and IMU sensors is critical to mitigate distortions and maintain map quality. Our proposed method leverages robust plane detection through voxel-based segmentation and a plane scoring approach, which effectively handles noise and edge disruptions, resulting in reliable plane identification even in complex indoor environments. By calibrating the initial poses based on these detected planes, the proposed method ensures low calibration errors and consistent performance, even with sensor motion. Through comparative evaluations, it is demonstrated that the proposed method outperforms existing calibration approaches in terms of accuracy and computational efficiency, making it well-suited for real-time, handheld applications. Our contributions to plane detection and calibration advance the potential for accurate localization and mapping in applications spanning architecture, engineering, and autonomous navigation in GPS-denied environments. Future work may explore further optimization for dynamic and outdoor settings, where varying lighting and environmental conditions pose additional challenges.

## Figures and Tables

**Table 1:** A taxonomy of existing LiDAR-IMU calibration methods.

Ref.	Year	Modality	Platform	Env	Approach	Limitations
[6]	2018	LiDAR+IMU	Handheld	Indoor	Scan-based	Precise calibration is not achievable due to the use of low-accuracy upsampled LiDAR points.
[7]	2021	LiDAR+IMU	Handheld	Indoor	Pre-define map	Requires a pre-defined map
[8]	2022	LiDAR+IMU	Handheld	Indoor	Scan-based	Since the first LiDAR scan is defined in the global frame, the pose in the first LiDAR scan affects the accuracy of the calibration
[9]	2024	LiDAR+IMU+Camera	Handheld	Indoor/Outdoor	Scan-based	Due to the two-step calibration process involving both the camera and LiDAR, a complex processing workflow is required, and the calibration accuracy of each sensor affects the accuracy of the other
[10]	2024	LiDAR+IMU	Vehicle	Outdoor	Ground information	Not suitable for handheld LiDAR
[11]	2024	Multi LiDAR+IMU	Vehicle	Indoor	IMU-based	Since the calibration is based on IMU data, it is sensitive to IMU drift and noise
[12]	2020	LiDAR+IMU	Handheld	Indoor/Outdoor	No initial pose estimation	High cost of computation
[13]	2023	LiDAR+IMU	Handheld, Vehicle	Indoor/Outdoor	No initial pose estimation	High cost of computation

**Table 2:** Notations and their explanation.

Notations	Explanations
$P,\;p_{l}$	The set of LiDAR data and the *l*-th point
$V_{W,H,C}$	A voxel containing (W×H×C) elements
$v_{w,h,c}$	The voxel located at (w,h,c)
$P_{w,h,c}^{\prime }\in R^{3},p_{v_{w,h,c}}^{i}\in R^{3}$	The LiDAR total points $v_{w,h,c}$ at (w,h,c), and the *i*-th point inside the voxel $v_{w,h,c}$
$n_{j}\in R^{3},\;\bar{n}\in R^{3}$	The *j*-th normal vector and the mean vector within the voxel
${\;U}_{Q}$	The final *Q* detected planes
${\;u}_{q}$	The *q*-th plane detected through the proposed method
$\begin{array}{c} R_{init}\in SO\left(3\right),\;n_{gt}\in R^{3},n_{avg}\in R^{3} \end{array}$	The initial rotation matrix, the normal vector in the global frame, and the normal vector in the LiDAR frame
RLG∈SO(3)	The rotation matrix between the global and LiDAR frames
RIG∈SO(3)	The rotation matrix between the global and IMU frames
$n_{G}\in R^{3}$	The normal vector of the actual plane

**Table 3:** LiDAR sensor details in SLAM datasets.

Dataset	Sensor	Frame Rate	Horizontal FOV	Vertical FOV	Maximum Detection Range	Detection Distance Accuracy
LiDAR-Net [16]	Leica BLK2GO	5 Hz	210°	85°	10 m	≤5 mm
Hilti [17]	Ouster OSO-64	10 Hz	360°	90°	50 m	≤5 cm
VECtor [18]	Ouster OSO-128	20 Hz	360°	90°	50 m	≤5 cm

**Table 4:** IMU sensor details in SLAM datasets.

Dataset	Sensor	Frame Rate	Accelerometer	Gyroscope	Magnetometer
Hilti [17]	InvenSense ICM-20948	100 Hz	√	√	√
VECtor [18]	XSens MTi-30 AHRS	200 Hz	√	√	√

**Table 5:** Performance evaluation of the proposed method using different values of $d_{thresh}$ on the corridor sequence of LiDAR-Net.

$d_{\mathrm{thresh}}$	F1 Score			Time		
	σ=0.00	σ=0.03	σ=0.05	σ=0.00	σ=0.03	σ=0.05
0.0	83.3	79.5	78.5	0.83	1.00	1.07
0.1	90.6	81.2	80.3	0.85	**1.00**	**1.07**
**0.2**	**91.4**	**83.2**	**81.8**	**0.85**	1.02	1.09
0.4	89.7	79.8	79.2	0.85	1.02	1.09
0.6	87.0	79.6	78.5	0.87	1.02	1.09
0.8	85.5	77.3	75.5	0.87	1.03	1.10
1.0	79.8	76.3	74.2	0.87	1.03	1.10

**Table 6:** Performance evaluation of the proposed method using different values of $s_{thresh}$ on the corridor sequence of LiDAR-Net.

$s_{\mathrm{thresh}}$	F1 Score			Time		
	σ=0.00	σ=0.03	σ=0.05	σ=0.00	σ=0.03	σ=0.05
0.0	87.5	79.2	78.6	0.85	1.00	1.07
0.1	91.1	81.2	79.2	0.86	**1.00**	**1.07**
**0.2**	**91.4**	**83.2**	**81.8**	**0.85**	1.02	1.08
0.3	91.2	80.8	79.8	0.85	1.02	1.08
0.4	90.1	78.5	78.7	0.85	1.02	1.08
0.5	89.3	75.8	72.4	0.86	1.03	1.09
0.6	86.9	72.3	69.2	0.86	1.03	1.09
0.7	79.9	71.2	60.8	0.86	1.04	1.09
0.8	78.4	69.5	45.9	0.86	1.04	1.10
0.9	56.2	52.2	30.3	0.87	1.05	1.11
1.0	43.7	28.9	24.2	0.87	1.06	1.13

**Table 7:** Comparison of the proposed method with benchmark plane detection algorithms on the LiDAR-Net dataset.

Method	Corridor				Room			
	Precision (%)	Recall (%)	F1-Score (%)	Time (s)	Precision (%)	Recall (%)	F1-Score(%)	Time (s)
RANSAC [19]	89.9	72.4	80.2	2.6	**96.9**	57.8	72.4	4.9
3D-Hough [21]	**99.0**	48.9	65.4	13.5	90.9	40.9	56.4	23.9
Region Growing [20]	95.1	79.6	86.7	183.7	87.4	60.8	71.7	339.6
Ours	90.5	**92.3**	**91.4**	**0.85**	93.9	**78.8**	**85.7**	**2.34**

**Table 8:** Performance comparison with SOTA methods in terms of the RMSE (Roll and pitch) on the Hilti and VECtor datasets.

Method	Hilti (Basement)		VECtor (Corridor)	
	RMSE (Deg)		RMSE (Deg)	
	Roll	Pitch	Roll	Pitch
LI-init [8]	0.73	0.41	21.8	26.7
Gril-calib [10]	**0.21**	0.07	10.5	18.3
Proposed method	0.41	**0.06**	**3.8**	**1.4**

## Data Availability

Data are contained within the article.
